# Multimodal transformer and directed relational graph learning for emotion recognition in conversations with potential applications in college student mental health research

**DOI:** 10.3389/fpsyg.2026.1867034

**Published:** 2026-09-14

**Authors:** Jing Zhao, Feng Liu

**Affiliations:** 1 Anhui Communications Vocational & Technical College, Hefei, China; 2 Hefei Longchi Quantum Techology Co., Ltd, Hefei, China

**Keywords:** affective computing, cross-modal attention, graph neural network, multimodal conversational emotion recognition, relational graph learning, college student mental health

## Abstract

Multimodal emotion recognition in conversations aims to identify the emotion expressed in each utterance by jointly analyzing textual, acoustic, visual, and contextual information. However, existing methods still face challenges in coordinating intra-modal dependency modeling, cross-modal interaction, and graph-based conversational reasoning. To address these challenges, this paper proposes MTG-ERC, a multimodal conversational emotion recognition framework that integrates transformer-based attention mechanisms with directed relational graph learning. First, textual, acoustic, and visual features are projected into a unified representation space. Intra-modal self-attention and cross-modal attention are then employed to capture modality-specific contextual dependencies and complementary interactions among modalities. Subsequently, a directed multi-relational graph module combines relational graph convolution and Graph Transformer operations to model temporal context, speaker-related interactions, and multimodal dependencies among utterances. Finally, global attention-based representations and local graph-based contextual representations are integrated for utterance-level emotion classification. Experiments on the IEMOCAP and MELD datasets show that MTG-ERC achieves competitive performance compared with the evaluated conversational emotion recognition baselines. Ablation results further indicate that intra-modal attention, cross-modal interaction, and directed relational modeling make complementary contributions to the final performance. The proposed framework also has potential applications in college student mental health research, where its multimodal affective modeling capability could support the analysis of emotional patterns in conversational data.

## 1 Introduction

Emotion recognition in conversations aims to identify the emotional state expressed in each utterance by considering both multimodal cues and conversational context. It is an important research problem in affective computing because emotions are often conveyed jointly through semantic content, vocal characteristics, facial expressions, speaker interactions, and preceding conversational information. Accurate conversational emotion recognition can support human−computer interaction, dialogue systems, educational technologies, and other emotion-aware applications, and may also have value in college student mental health research by supporting the analysis of emotional patterns in student conversations.

Early emotion recognition methods mainly relied on a single modality, such as text, speech, or facial expressions. However, unimodal information is often incomplete or ambiguous. Text may provide explicit semantic content but may not fully reflect tone or emotional intensity. Acoustic features can capture pitch, speaking rate, and energy, whereas visual features provide facial and behavioral cues. Multimodal emotion recognition therefore combines complementary information from textual, acoustic, and visual modalities to obtain more comprehensive emotional representations.

Recent deep learning methods have substantially improved multimodal conversational emotion recognition. Attention-based architectures can model long-range dependencies and interactions among modalities, while graph neural networks can represent speaker relationships and contextual connections among utterances. Nevertheless, several challenges remain. First, modality-specific emotional patterns and cross-modal interactions are often modeled separately, making it difficult to coordinate intra-modal and inter-modal information. Second, conversational emotions depend on both local transitions and broader contextual relationships, which cannot always be adequately represented by sequential or fully connected structures. Third, multimodal graph construction may introduce redundant connections and increased computational cost if temporal direction and relation types are not explicitly controlled.

To address these issues, this paper proposes MTG-ERC, a multimodal conversational emotion recognition framework that integrates attention-based representation learning with directed multi-relational graph modeling. The framework uses intra-modal self-attention to capture modality-specific contextual dependencies and cross-modal attention to exchange complementary emotional information among text, audio, and visual modalities. It then constructs a directed relational graph in which modality nodes and temporally neighboring utterances are connected through relation-specific edges. Relational graph convolution and Graph Transformer operations are further employed to aggregate local multimodal interactions and conversational dependencies. Finally, the attention-based global representations and graph-based contextual representations are integrated for emotion classification.

The main contributions of this study are summarized as follows:

*A coordinated attention–graph framework is developed for multimodal conversational emotion recognition*. The framework combines transformer-based global dependency modeling with graph-based local relational reasoning instead of relying on either sequential attention or graph convolution alone.

*An intra-modal and cross-modal attention module is designed to preserve modality-specific emotional patterns while enabling complementary information exchange among textual, acoustic, and visual representations*. A gating mechanism is further used to reduce irrelevant cross-modal information.

*A directed multi-relational graph module is constructed to model both within-utterance multimodal relations and temporal dependencies among neighboring utterances*. Relation-specific graph convolution and Graph Transformer operations are jointly used to obtain contextual representations, which are subsequently fused with the attention-based representations for utterance-level emotion classification.

Although conversational emotion recognition may provide affective information for future human-centered support systems, the present study does not evaluate psychological disorders, mental health risks, or clinical outcomes. Therefore, the proposed method should be interpreted as an emotion recognition framework rather than a psychological assessment or diagnostic system.

The remainder of this paper is organized as follows. Section 2 reviews studies on multimodal emotion recognition, attention-based fusion, and graph-based conversational modeling. Section 3 presents the proposed MTG-ERC framework. Section 4 introduces the experimental settings and reports the comparative, statistical, ablation, efficiency, and error-analysis results. Section 5 discusses the main findings, limitations, and ethical considerations. Section 6 concludes the paper.

## 2 Related work

This section reviews the literature most closely related to the proposed MTG-ERC framework, including multimodal emotion recognition and benchmark resources, attention-based multimodal fusion, graph-based conversational reasoning, and the research gap addressed in this study.

### 2.1 Multimodal emotion recognition and benchmark resources

Emotion recognition has become an important research topic in affective computing and human-centered artificial intelligence. Early studies mainly relied on unimodal signals such as text, speech, or facial expressions, which often failed to capture the complexity of human affective expressions. With the release of benchmark corpora such as IEMOCAP, EmotionLines, and MELD, multimodal emotion recognition, especially emotion recognition in conversations, has developed rapidly. IEMOCAP provides acted dyadic interactions with acoustic, visual, and motion information; EmotionLines extends dialogue-level emotion annotation to conversational text; and MELD supports multi-party multimodal conversational emotion recognition using textual, acoustic, and visual information (Busso et al., 2008; Hsu and ku, 2018; Poria et al., 2019).

Recent surveys have shown that multimodal emotion recognition systems increasingly rely on the joint modeling of textual, acoustic, and visual information because different modalities provide complementary emotional evidence. Reviews by Lian et al. (2023); Fu et al. (2023); Wu et al. (2025b) indicate that the field has gradually shifted from simple feature concatenation toward structured multimodal interaction modeling, with increasing attention to robustness, temporal dynamics, speaker dependencies, and conversational context. Hazmoune and Bougamouza (2024) further reviewed transformer-based multimodal emotion recognition methods and summarized existing studies according to their model architectures, fusion strategies, and application settings. Separately, affective computing and machine learning methods have also been explored in student mental health and other psychologically relevant scenarios (Drira et al., 2024; Chen and Zhong, 2025). However, these application-oriented studies employ different labels, populations, and validation objectives from benchmark conversational emotion recognition and do not provide direct evidence that an ERC model can perform psychological assessment.

### 2.2 Attention-based multimodal fusion and contextual modeling

Attention-based architectures have played an important role in multimodal representation learning. The Multimodal Transformer (MulT) introduced directional pairwise cross-modal attention for unaligned multimodal sequences and became a foundational model for subsequent cross-modal interaction studies (Tsai et al., 2019). Later methods, including MISA and Self-MM, emphasized the importance of learning modality-invariant or shared representations together with modality-specific information, thereby reducing the heterogeneity gap among modalities (Hazarika et al., 2020; Yu et al., 2021). These studies demonstrate that effective multimodal fusion should not merely concatenate heterogeneous features but should explicitly model their shared, complementary, and modality-specific information.

In conversational settings, attention mechanisms are often combined with contextual modeling to capture speaker states, inter-utterance dependencies, and emotion shifts. Representative methods such as DialogueRNN, DialogXL, and DialogueCRN improve ERC performance by modeling speaker-aware memory, long-context dependencies, or cognitive-style contextual reasoning (Majumder et al., 2019; Shen et al., 2021a; Hu et al., 2021a). For multimodal ERC, MultiEMO employs bidirectional multi-head cross-attention to capture correlation-aware interactions among textual, acoustic, and visual modalities, demonstrating the importance of explicit cross-modal information exchange (Shi and Huang, 2023). Nevertheless, attention-based fusion alone does not explicitly represent relation-specific connections among speakers, modalities, and temporally neighboring utterances. This limitation motivates the integration of attention-based global dependency modeling with structured graph-based relational reasoning.

### 2.3 Graph neural networks and relational reasoning for conversational emotion recognition

Graph neural networks have become an important modeling paradigm for conversational emotion recognition because they can represent speaker interactions, long-distance dependencies, and structural relationships among utterances. DialogueGCN demonstrated that self-speaker and inter-speaker dependencies can be represented through relation-aware graph convolution (Ghosal et al., 2019). MMGCN subsequently extended graph modeling to multimodal conversational representations (Hu et al., 2021b), while DAG-ERC reformulated contextual information propagation through a directed acyclic graph to improve long-range conversational modeling (Shen et al., 2021b). COSMIC incorporated commonsense knowledge into contextual reasoning (Ghosal et al., 2020), and GraphCFC introduced directed graph-based cross-modal feature complementation to reduce redundant multimodal interactions and information loss (Li et al., 2024).

More recent studies have explored adaptive graph construction, higher-order relational learning, dynamic contextual modeling, and multimodal knowledge integration. Adaptive Graph Learning employs adaptive node and edge selection to improve intra-modal and cross-modal interactions (Tu et al., 2024). MultiDAG+CL combines directed acyclic graph learning with curriculum learning to address emotion shifts and class imbalance (Nguyen et al., 2024). The Multiple Knowledge-Enhanced Interactive Graph Network incorporates textual and visual commonsense knowledge into multimodal relational reasoning (Tu et al., 2024). Other studies have investigated graph-spectrum modeling to distinguish consistent and complementary multimodal information (Meng et al., 2024), neural ordinary differential equation-based dynamic graph modeling to capture continuous emotional changes (Shou et al., 2025), diffusion-based learning to reduce cross-modal noise and redundancy (Tian et al., 2025), and evidence–cause attention to distinguish direct emotional evidence from contextual emotional causes (Zhang and Tan, 2025). A recent comprehensive review further summarizes current multimodal emotion recognition techniques, fusion mechanisms, challenges, and future directions (Wu et al., 2025a). More recently, Cross-Space Synergy has explored high-order cross-modal interactions and training stability through polynomial representation fusion and multi-objective gradient optimization (Lyu et al., 2026).

These developments indicate that current multimodal ERC research is moving beyond conventional feature concatenation and shallow graph convolution toward more adaptive, knowledge-enhanced, interaction-aware, and optimization-aware architectures. Separately, multimodal affect analysis has also been investigated in student mental health and psychologically informed systems (Drira et al., 2024; Chen and Zhong, 2025). However, these studies use different outcome variables and validation settings from IEMOCAP and MELD. Their findings therefore cannot be directly used to establish that conversational emotion recognition models are valid tools for psychological assessment.

### 2.4 Research gap and positioning of MTG-ERC

Existing conversational emotion recognition methods mainly rely on attention-based multimodal fusion or graph-based contextual reasoning. Attention mechanisms are effective in modeling modality-specific and cross-modal dependencies, whereas graph neural networks explicitly represent speaker interactions and contextual relationships among utterances. However, the coordinated modeling of intra-modal information, controlled cross-modal interaction, and relation-specific conversational context remains insufficient.

DialogueGCN mainly models speaker and temporal relations through relational graph convolution (Ghosal et al., 2019), while MMGCN extends graph learning to multimodal conversational representations (Hu et al., 2021b). DAG-ERC focuses on directed contextual propagation (Shen et al., 2021b), and MultiEMO emphasizes bidirectional cross-modal attention without a separate relational graph reasoning stage (Shi and Huang, 2023). GraphCFC introduces directed graph-based cross-modal feature complementation (Li et al., 2024). In comparison, MTG-ERC combines an intra-transformer for modality-specific dependency modeling, an inter-transformer with gating for cross-modal information exchange, and a directed multi-relational graph for modeling within-utterance multimodal relations and temporal contextual dependencies. The resulting attention-based and graph-based representations are subsequently integrated for utterance-level emotion classification.

Therefore, the distinction of MTG-ERC lies not in introducing attention or graph neural networks individually, but in coordinating intra-modal attention, gated cross-modal interaction, relation-aware graph convolution, and Graph Transformer refinement within a unified framework. Although Hazmoune and Bougamouza reported an accuracy of 86.3% for an IEMOCAP six-class setting (Hazmoune and Bougamouza, 2024), results across ERC studies may differ because of variations in data partitions, feature extractors, pretrained representations, and evaluation protocols. MTG-ERC is therefore positioned as a competitive method under the adopted experimental setting rather than as a universally state-of-the-art model. Moreover, because IEMOCAP and MELD do not contain clinical psychological labels, the present study should be interpreted as conversational emotion recognition rather than psychological assessment.

## 3 Methods

This section describes the proposed MTG-ERC framework, including its overall architecture, unimodal feature extraction, attention- and graph-based multimodal fusion mechanisms, and implementation details. The mathematical formulations used for model definition, evaluation metrics, and computational complexity analysis are presented in Equations 1−32.

### 3.1 Overall framework

This study proposes MTG-ERC, a multimodal conversational emotion recognition framework that combines attention-based multimodal representation learning with directed relational graph modeling. Given a conversation containing textual, acoustic, and visual information, the model predicts an emotion label for each utterance.

As illustrated in Figure 1, MTG-ERC consists of four components: unimodal feature extraction, attention-based multimodal fusion, directed multi-relational graph modeling, and emotion classification. First, textual, acoustic, and visual features are extracted and projected into a shared representation space. Second, an intra-transformer models contextual dependencies within each modality, while an inter-transformer captures complementary interactions across modalities. Third, a directed multi-relational graph is constructed to represent within-utterance multimodal relations and temporal dependencies among neighboring utterances. Relational graph convolution and Graph Transformer operations are then used to obtain graph-based contextual representations. Finally, the attention-based global representations and graph-based contextual representations are integrated for utterance-level emotion classification.

**Figure 1:**
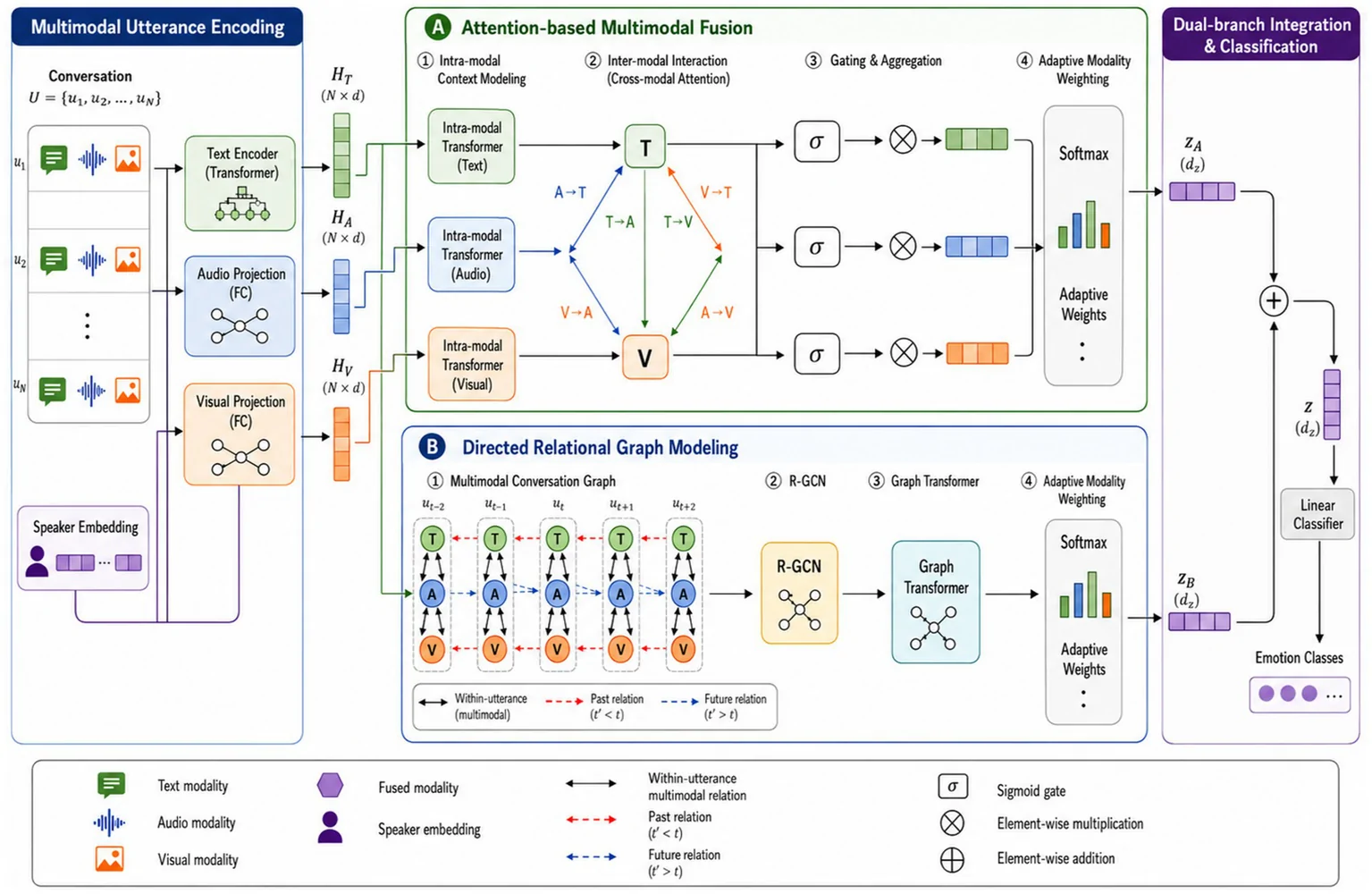
Overall architecture of the proposed MTG-ERC framework.

The proposed framework is designed for conversational emotion recognition. Because the datasets used in this study do not contain clinical psychological labels, the framework is not intended to perform psychological diagnosis or formal mental health assessment.

### 3.2 Unimodal feature extraction

Each conversation is represented as

$$C=\{u_{1},u_{2},\ldots ,u_{N}\},$$ (1)

where N denotes the number of utterances in the conversation. For the i -th utterance, the textual, acoustic, and visual features are denoted by $x_{i}^{t}$ , $x_{i}^{a}$ , and $x_{i}^{v}$ , respectively.

Because the original feature dimensions differ across modalities and datasets, modality-specific projection layers are used to map all features into a common 128-dimensional representation space:

$$h_{i}^{m}=\mathrm{LN}(W_{m}x_{i}^{m}+b_{m}),m\in \{t,a,v\},$$ (2)

where $W_{m}$ and $b_{m}$ are trainable parameters, m denotes the modality, and LN(·) denotes layer normalization.

#### 3.2.1 Textual features

Textual information provides the semantic content of each utterance. For IEMOCAP, Sentence-BERT is used to obtain a 768-dimensional sentence-level representation. For MELD, RoBERTa-Large is used to obtain a 1,024-dimensional utterance-level representation. The hidden representation corresponding to the first token is used as the initial textual feature for MELD.

The textual features are projected into the common representation space as

$$h_{i}^{t}=\mathrm{LN}(W_{t}x_{i}^{t}+b_{t}),$$ (3)

where $x_{i}^{t}$ is the pre-extracted textual feature and $h_{i}^{t}\in {\mathbb{R}}^{128}$ is the projected textual representation.

Sentence-BERT and RoBERTa-Large are used as frozen feature extractors. Their parameters are not updated during MTG-ERC training. Only the modality projection layer and subsequent attention, graph, and classification modules are optimized. This strategy reduces training cost and ensures that the feature extraction procedure remains consistent across experimental runs.

#### 3.2.2 Acoustic features

Acoustic information provides paralinguistic cues such as pitch, intensity, speaking rate, jitter, and shimmer. OpenSmile is used to extract utterance-level acoustic features.

For IEMOCAP, the GeMAPS configuration is used to obtain a 100-dimensional acoustic representation. For MELD, the ComParE configuration is adopted to obtain a 300-dimensional acoustic representation because MELD contains more complex acoustic environments and background noise.

The acoustic feature is projected into the common representation space as

$$h_{i}^{a}=\mathrm{LN}(\text{ReLU}(W_{a}x_{i}^{a}+b_{a})),$$ (4)

where $x_{i}^{a}$ is the original acoustic feature and $h_{i}^{a}\in {\mathbb{R}}^{128}$ is the projected acoustic representation.

A dropout layer with a rate of 0.30 is applied after the projection layer to reduce overfitting.

#### 3.2.3 Visual features

Visual information includes facial expressions, facial action units, head pose, and other behavioral cues. For IEMOCAP, OpenFace is used to extract frame-level facial landmarks, facial action units, and head-pose information. Mean temporal pooling is then applied to obtain a 512-dimensional utterance-level visual representation.

For MELD, a pretrained DenseNet is used to extract frame-level visual features. Mean temporal pooling over the valid video frames produces a 342-dimensional utterance-level visual representation.

The visual feature is projected into the shared representation space as

$$h_{i}^{v}=\mathrm{LN}(\text{ReLU}(W_{v}x_{i}^{v}+b_{v})),$$ (5)

where $x_{i}^{v}$ is the original visual feature and $h_{i}^{v}\in {\mathbb{R}}^{128}$ is the projected visual representation.

A dropout rate of 0.30 is also applied after visual feature projection. The projected feature sequence for modality m is represented as

$$H^{m}=[h_{1}^{m},h_{2}^{m},\ldots ,h_{N}^{m}],m\in \{t,a,v\}.$$ (6)

### 3.3 Attention-based and graph-based multimodal fusion

This subsection describes the core multimodal fusion and contextual reasoning mechanisms of MTG-ERC, including speaker-aware representation, intra-modal and cross-modal attention, directed multi-relational graph construction, graph-based contextual modeling, and final multimodal fusion.

#### 3.3.1 Task formulation and speaker embedding

The objective of conversational emotion recognition is to predict the emotion label $y_{i}$ of each utterance $u_{i}$ from a predefined emotion set Y :

$${\hat{y}}_{i}=\arg\max_{y\in Y}p(y,|\mathrm{Ci}).$$ (7)

Because speaker identity affects emotional expression and contextual interpretation, a learnable embedding is assigned to each speaker. Let $q_{i}$ denote the speaker of utterance $u_{i}$ , and let $e_{q_{i}}\in {\mathbb{R}}^{128}$ denote the corresponding speaker embedding. The modality representation is enhanced as

$${\tilde{h}}_{i}^{m}=h_{i}^{m}+\lambda_{s}e_{q_{i}},$$ (8)

where $\lambda_{s}$ is a learnable scalar initialized to 1.0. The speaker embeddings are randomly initialized and jointly optimized with the complete network.

For conversations containing different numbers of speakers, each speaker is assigned an index within the corresponding conversation. Padding positions are excluded from attention calculation, graph aggregation, loss computation, and metric calculation through a binary utterance mask.

#### 3.3.2 Intra-modal and cross-modal attention

The attention-based fusion module contains an intra-transformer and an inter-transformer, as illustrated in Figure 2.

**Figure 2:**
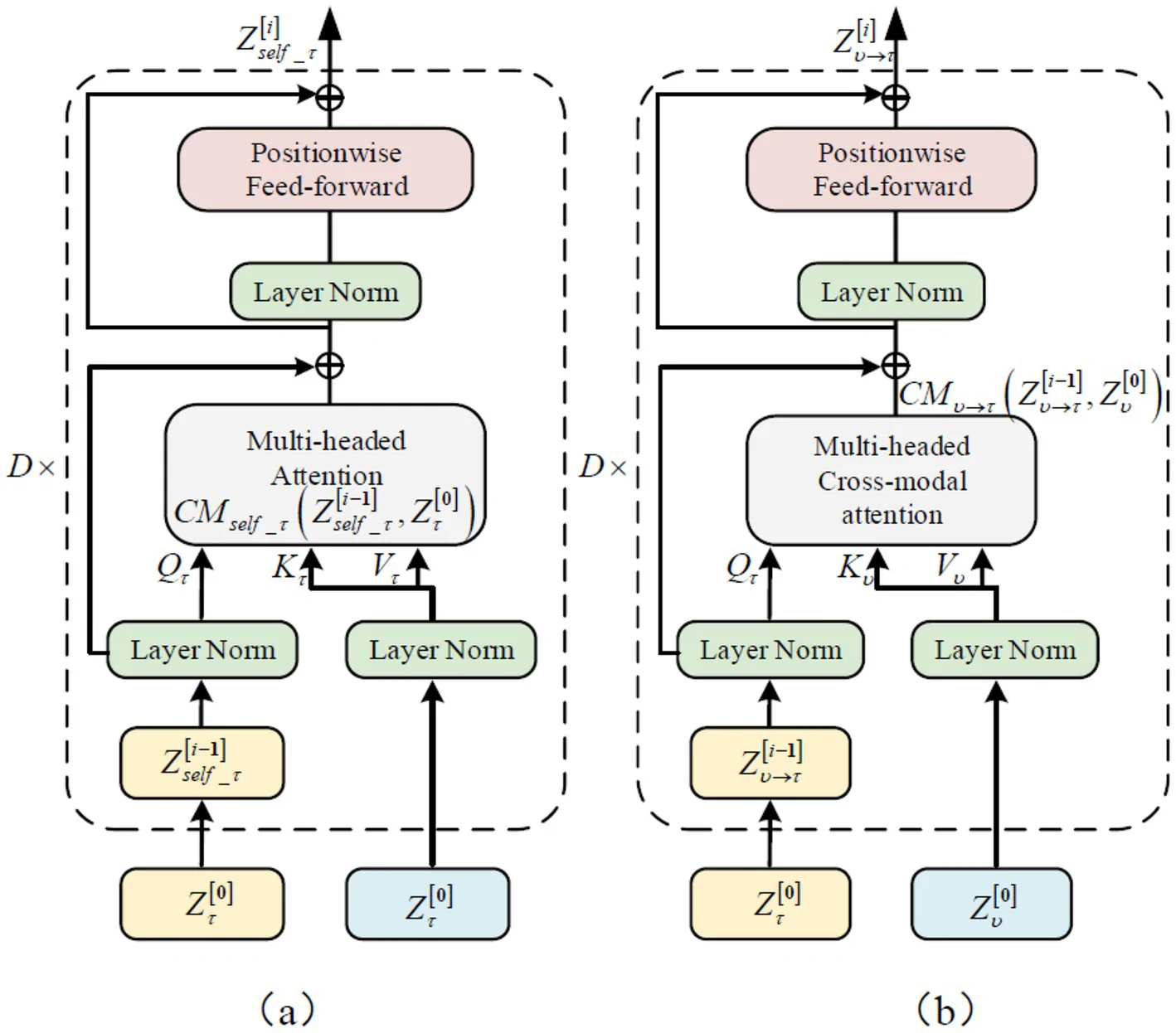
Structures of the attention modules: **(a)** intra-transformer for modality-specific contextual modeling and **(b)** inter-transformer for gated cross-modal interaction.

The intra-transformer models contextual dependencies within each modality. For modality m , the speaker-enhanced sequence is processed as

$${\bar{H}}^{m}={\mathrm{Trm}}_{\text{intra}}^{m}({\tilde{H}}^{m}),m\in \{t,a,v\},$$ (9)

where ${\mathrm{Trm}}_{\text{intra}}^{m}$ denotes the modality-specific transformer encoder.

Each intra-transformer contains two encoder layers. Each layer uses four attention heads, a hidden dimension of 128, and a feed-forward dimension of 512. Residual connections and layer normalization are applied after the multi-head self-attention and feed-forward operations. A dropout rate of 0.30 is used in the attention, feed-forward, and residual branches.

The inter-transformer models directed information exchange between different modalities. For a target modality m and an auxiliary modality n , cross-modal attention is calculated as

$$C^{m\leftarrow n}=\mathrm{MHA}(Q,=,{\bar{H}}^{m}K={\bar{H}}^{n}V={\bar{H}}^{n}),$$ (10)

where $C^{m\leftarrow n}$ represents information transferred from modality n to modality m .

A gating mechanism is used to reduce irrelevant cross-modal information:

$$G^{m\leftarrow n}=\sigma (W_{g}[{\bar{H}}^{m}∥C^{m\leftarrow n}]+b_{g}),$$

$$Z^{m\leftarrow n}=G^{m\leftarrow n}⊙C^{m\leftarrow n}+(1-G^{m\leftarrow n})⊙{\bar{H}}^{m},$$ (11)

where σ(·) denotes the sigmoid function, ⊙ denotes element-wise multiplication, and ∥ denotes feature concatenation.

For each target modality, the representations obtained from the other two modalities are combined with the intra-modal representation:

$$Z^{m}=W_{f}[{\bar{H}}^{m}∥Z^{m\leftarrow n_{1}}∥Z^{m\leftarrow n_{2}}]+b_{f}.$$ (12)

Each inter-transformer contains two cross-modal attention layers with four attention heads. The query, key, and value dimensions are all set to 128, and the feed-forward dimension is set to 512. A dropout rate of 0.30 is applied after the cross-modal attention and feed-forward operations.

#### 3.3.3 Directed multi-relational graph construction

To capture structured conversational relationships, a directed graph is constructed for each conversation:

G=(V,E,R), (13)

where V , E , and R denote the node set, edge set, and relation-type set, respectively.

For every utterance $u_{i}$ , three modality-specific nodes are constructed:

$$v_{i}^{t},v_{i}^{a},v_{i}^{v},$$ (14)

corresponding to the textual, acoustic, and visual modalities. Their initial node features are obtained from $Z_{i}^{t}$ , $Z_{i}^{a}$ , and $Z_{i}^{v}$ , respectively.

Two groups of relations are considered. First, within-utterance edges connect the three modality nodes belonging to the same utterance. Directed modality-to-modality connections and self-loops produce nine relation types:

$$R_{\text{cross}}=\{\begin{array}{l} t,\to ,t,t,\to ,a,t,\to ,\mathrm{va}\to \mathrm{ta}\to \\ \mathrm{aa}\to \mathrm{vv}\to \mathrm{tv}\to \mathrm{av}\to v \end{array}\}.$$ (15)

Second, temporal edges connect nodes of the same modality across neighboring utterances. For each modality, separate relation types are defined for past-to-current and future-to-current information. These connections produce six temporal relation types:

$$R_{\text{temporal}}=\{t_{p},\to ,t,t_{f},\to ,ta_{p}\to aa_{f}\to av_{p}\to vv_{f}\to v\},$$ (16)

where the subscripts p and f denote past and future utterances, respectively.

To avoid the computational cost of a fully connected graph, each current utterance is connected only to the 10 preceding utterances and 10 following utterances. The temporal neighborhood is therefore defined as

j∈[max,(1,i−10)min(N,i+10)]. (17)

Thus, the contextual graph window is set to P=10 and F=10 . For utterances near the beginning or end of a conversation, only the available neighboring utterances are used.

The graph contains 15 relation types in total: nine within-utterance modality relations and six modality-specific temporal relations.

#### 3.3.4 Relational graph convolution and graph transformer

A relational graph convolutional network is first applied to aggregate relation-specific neighborhood information. For node p , the representation at layer l+1 is calculated as

$$r_{p}^{\left(l+1\right)}=\sigma (W_{0}^{\left(l\right)}r_{p}^{\left(l\right)}+\sum_{r\in R}\;\sum_{q\in N_{r}(p)}\frac{1}{c_{p,r}}W_{r}^{\left(l\right)}r_{q}^{\left(l\right)}),$$ (18)

where $N_{r}(p)$ denotes the neighboring nodes of node p under relation r , $c_{p,r}$ is the number of neighbors under relation r , and $W_{r}^{\left(l\right)}$ is the relation-specific transformation matrix.

The RGCN contains two layers with a hidden dimension of 128. To reduce the number of relation-specific parameters, basis decomposition is used with five basis matrices. ReLU activation and a dropout rate of 0.30 are applied after each RGCN layer.

The RGCN output is subsequently processed by a Graph Transformer. For attention head k , the attention coefficient between nodes p and q is

$$\alpha_{\mathrm{pq}}^{k}=\text{soft}\max_{q}(\frac{{\left(W_{Q}^{k}r_{p}\right)}^{Τ}(W_{K}^{k}r_{q})}{\sqrt{d_{k}}}).$$ (19)

The updated node representation is

$$g_{p}=\mathrm{LN}[r_{p}+{∥}_{k=1}^{K}\sum_{q\in N(p)}\alpha_{\mathrm{pq}}^{k}W_{V}^{k}r_{q}],$$ (20)

where K=4 is the number of graph-attention heads and $d_{k}=32$ is the representation dimension of each head.

The Graph Transformer contains two layers with four attention heads, a hidden dimension of 128, and a feed-forward dimension of 512. Residual connections, layer normalization, and a dropout rate of 0.30 are applied in each layer.

This two-stage graph module first distinguishes different relation types through the RGCN and then refines the importance of neighboring nodes through graph-based multi-head attention.

#### 3.3.5 Multimodal fusion and emotion classification

The attention module produces one contextual representation for each modality. A dynamic weighting mechanism estimates the contribution of the three modalities:

$$[\beta_{i}^{t},\beta_{i}^{a},\beta_{i}^{v}]=\text{softmax}(W_{\beta }[Z_{i}^{t}∥Z_{i}^{a}∥Z_{i}^{v}]+b_{\beta }).$$ (21)

The attention-based multimodal representation is calculated as

$$z_{i}=\beta_{i}^{t}Z_{i}^{t}+\beta_{i}^{a}Z_{i}^{a}+\beta_{i}^{v}Z_{i}^{v}.$$ (22)

The three graph-node representations corresponding to utterance $u_{i}$ are concatenated and projected into a 128-dimensional representation:

$$g_{i}=\text{ReLU}(W_{g}[g_{i}^{t}∥g_{i}^{a}∥g_{i}^{v}]+b_{g}).$$ (23)

The final utterance representation combines the attention-based global representation and graph-based contextual representation:

$$f_{i}=z_{i}∥g_{i}.$$ (24)

Because both $z_{i}$ and $g_{i}$ are 128-dimensional, $f_{i}$ has a dimension of 256. The fused representation is passed through a fully connected layer with 128 hidden units:

$$o_{i}=\text{ReLU}(W_{f}f_{i}+b_{f}).$$ (25)

A dropout rate of 0.30 is applied to $o_{i}$ . The final emotion probability distribution is calculated as

$${\hat{p}}_{i}=\text{softmax}(W_{c}o_{i}+b_{c}).$$ (26)

The predicted emotion label is

$${\hat{y}}_{i}=\arg\max_{k}{\hat{p}}_{i,k}.$$ (27)

The complete model is optimized using the cross-entropy loss:

$$ℒ=-\frac{1}{M}\sum_{i=1}^{M}\;\sum_{k=1}^{K_{c}}y_{i,k}\log{\hat{p}}_{i,k},$$ (28)

where M is the number of valid utterances in the batch, $K_{c}$ is the number of emotion classes, $y_{i,k}$ is the ground-truth indicator, and ${\hat{p}}_{i,k}$ is the predicted probability.

### 3.4 Implementation details

MTG-ERC was implemented in Python 3.10.13 using PyTorch 2.1.2 and CUDA 12.1. All experiments were conducted on a workstation equipped with an NVIDIA GeForce RTX 3090 GPU with 24 GB of memory, an Intel Core i9-12900K processor, and 64 GB of system memory.

The modality projection dimension and speaker embedding dimension were both set to 128. The intra-transformer and inter-transformer each contained two layers with four attention heads. The hidden dimension was 128, and the feed-forward dimension was 512. The RGCN and Graph Transformer each contained two layers. Five basis matrices were used for RGCN relation decomposition, and the Graph Transformer used four attention heads. The graph context window included 10 preceding utterances and 10 following utterances.

The model was trained using the AdamW optimizer with an initial learning rate of $1\times 10^{-4}$ , a weight decay of $1\times 10^{-5}$ , and optimizer parameters $\beta_{1}=0.9$ , $\beta_{2}=0.999$ , and $\epsilon =1\times 10^{-8}$ . The batch size was set to eight conversations. Gradients were clipped to a maximum norm of 5.0 to improve training stability.

The maximum number of training epochs was set to 30. A cosine annealing learning-rate schedule was used to gradually reduce the learning rate from $1\times 10^{-4}$ to $1\times 10^{-6}$ . Early stopping with a patience of eight epochs was applied according to the weighted F1-score on the validation set. A dropout rate of 0.30 was used in the modality projection layers, transformer layers, graph layers, and classification layer.

The validation accuracy and weighted F1-score recorded across training epochs were used to generate the convergence curves presented in Figures 3 and 4.

**Figure 3:**
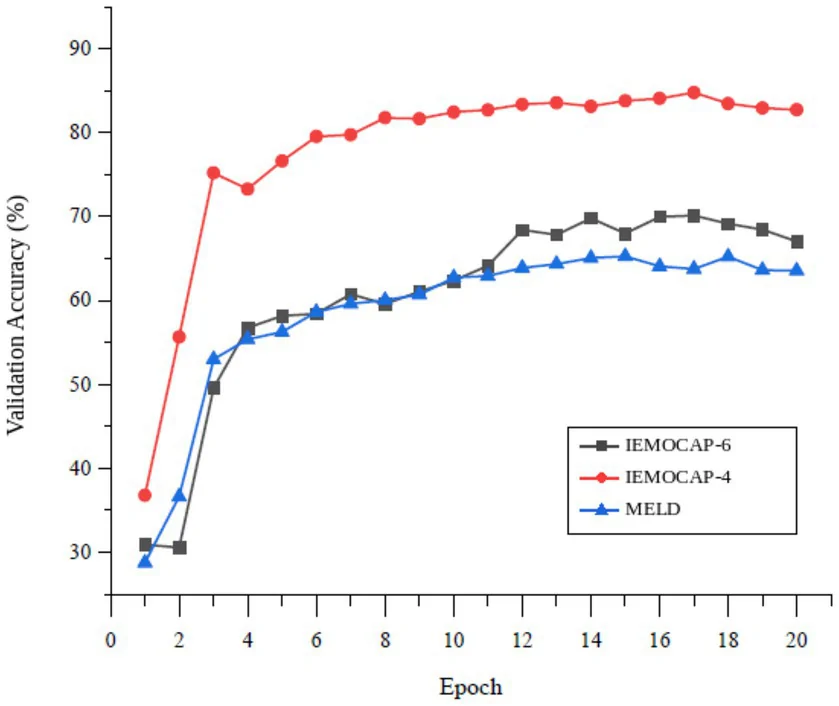
Validation accuracy curves of MTG-ERC on the IEMOCAP and MELD datasets.

**Figure 4:**
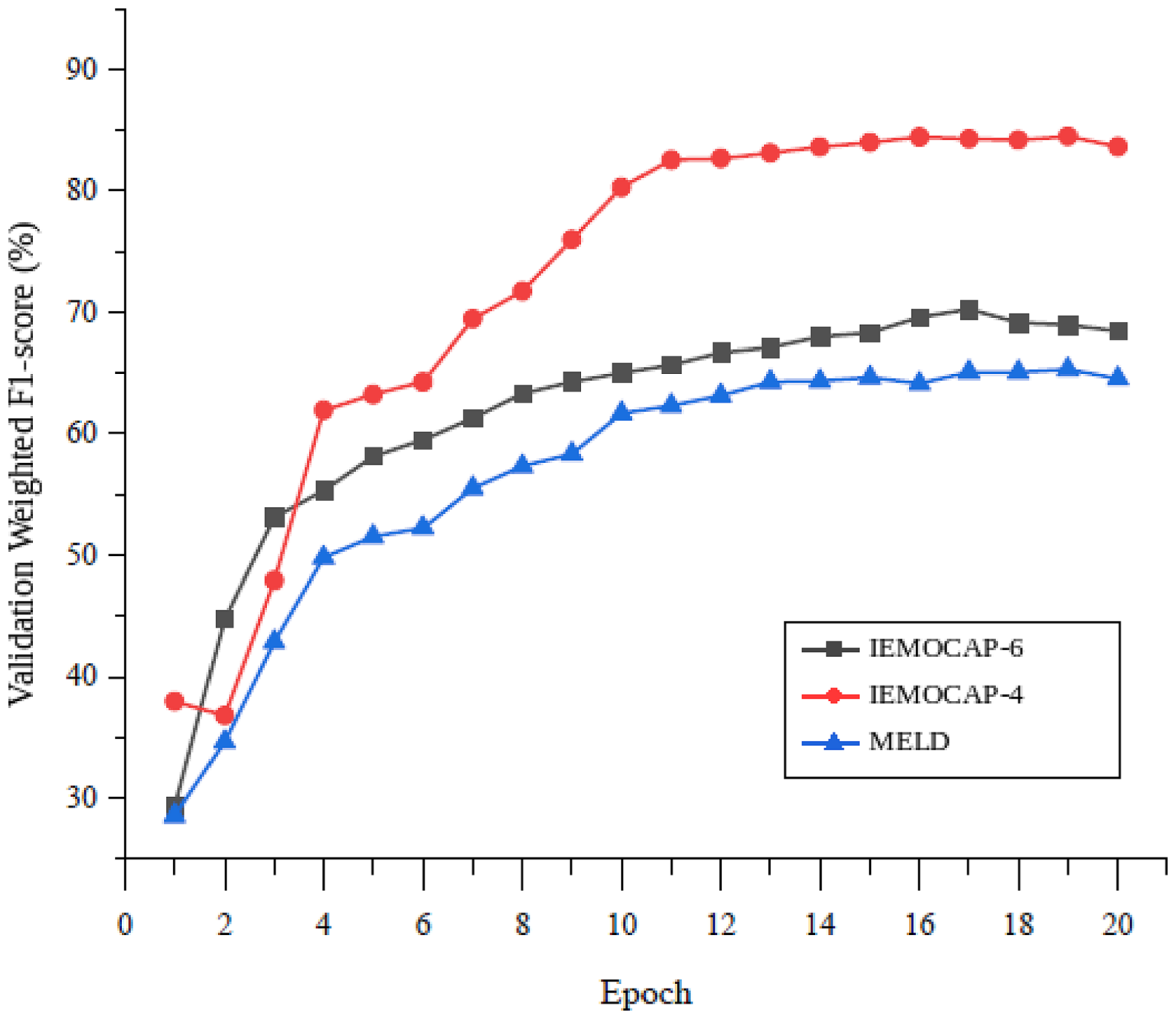
Validation weighted F1-score curves of MTG-ERC on the IEMOCAP and MELD datasets.

For IEMOCAP, the training and test partitions reported in Table 1 were used. 10% of the training conversations were randomly selected as the validation set, while the remaining training conversations were used for model optimization. The same training, validation, and test partitions were maintained across all random seeds to ensure a fair comparison.

**Table 1:** Statistics of the experimental datasets.

Dataset	Training/Test dialogues	Training/Test utterances
IEMOCAP, six-class	120/31	5,810/1,623
IEMOCAP, four-class	120/31	3,600/943
MELD	1,153/280	11,098/2,610

For MELD, the official training, validation, and test splits were used. Model selection was performed exclusively on the validation set, and the test set was used only for final evaluation.

To reduce the influence of random initialization, all experiments were independently repeated using five random seeds: 13, 21, 42, 87, and 100. For each run, the checkpoint with the highest validation weighted F1-score was selected and evaluated once on the corresponding test set. The final results are reported as the mean and standard deviation across the five runs.

Under the specified hardware configuration, one training run required approximately 38 min for the IEMOCAP six-class task, 31 min for the IEMOCAP four-class task, and 72 min for MELD. These times exclude the offline extraction of textual, acoustic, and visual features. The model generally converged within 17–23 epochs, although training was allowed to continue for a maximum of 30 epochs.

## 4 Results

This section presents the experimental evaluation of MTG-ERC, including the dataset and evaluation protocol, convergence analysis, comparison with existing methods, statistical analysis, ablation studies, computational efficiency, error analysis, and interpretation of the results.

### 4.1 Experimental datasets and evaluation protocol

The experimental protocol is described from two aspects: the benchmark datasets used for evaluation and the metrics adopted to quantify model performance.

#### 4.1.1 Experimental datasets

The proposed MTG-ERC framework was evaluated on two widely used multimodal conversational emotion recognition datasets, IEMOCAP and MELD.

IEMOCAP contains dyadic conversations collected across five sessions. Experiments were conducted under both six-class and four-class settings. The six-class setting includes happy, sad, neutral, angry, excited, and frustrated. In the four-class setting, excited is merged into happy, while frustrated is merged into sad.

MELD contains multi-party conversations derived from the television series *Friends*. It contains seven emotion categories: neutral, surprise, fear, sadness, joy, disgust, and anger. The official training, validation, and test partitions were used.

The dataset statistics are summarized in Table 1.

IEMOCAP represents acted dyadic interactions, whereas MELD contains more complex multi-party conversations. Together, the two datasets provide complementary evaluation settings. Nevertheless, evaluation on only two datasets limits the assessment of cross-domain generalizability.

#### 4.1.2 Evaluation metrics

Accuracy and weighted F1-score were adopted as the principal evaluation metrics. Accuracy is defined as

$$\mathrm{Acc}=\frac{\sum_{k=1}^{K}TP_{k}}{N},$$ (29)

where $TP_{k}$ denotes the number of correctly classified utterances in class k , K is the number of emotion classes, and N is the total number of evaluated utterances.

Because the emotion categories are imbalanced, the weighted F1-score was calculated as

$$w-F1=\sum_{k=1}^{K}\frac{n_{k}}{N}F1_{k},$$ (30)

where $n_{k}$ denotes the number of utterances belonging to class k , and $F1_{k}$ denotes the corresponding class-level F1-score.

To evaluate statistical robustness, each model was independently trained using five random seeds: 13, 21, 42, 87, and 100. The same training, validation, and test partitions were used for all random seeds. Results are reported as mean ± standard deviation across the five runs. Paired t -tests were performed on the matched seed-level results. A two-sided p<0.05 was regarded as statistically significant, and Cohen’s $d_{z}$ was used to report the paired effect size.

### 4.2 Training and convergence analysis

Figures 3 and 4 present the validation accuracy and validation weighted F1-score, respectively, across training epochs for the IEMOCAP six-class, IEMOCAP four-class, and MELD tasks. Performance improves rapidly during the early training stage and then gradually approaches a plateau. Only relatively small changes are observed during the later epochs. This convergence pattern is consistent with the use of validation-based early stopping to avoid unnecessary additional training once further performance improvement becomes limited. However, the presented validation curves alone do not provide direct evidence that early stopping prevents overfitting.

The IEMOCAP four-class task exhibits a comparatively smoother convergence trajectory than the six-class task. This pattern may partly reflect the reduced label ambiguity associated with merging closely related emotion categories, although the convergence curves alone do not isolate this factor. MELD shows somewhat greater epoch-to-epoch variation. Differences in conversational structure, class distribution, and multimodal data characteristics may contribute to this behavior; however, these possible factors were not independently evaluated in the present convergence analysis.

### 4.3 Comparison with existing methods

The comparative evaluation is reported separately for the IEMOCAP six-class task, the IEMOCAP four-class task, and the MELD dataset to examine model performance under different conversational and emotion-classification settings.

#### 4.3.1 Results on the IEMOCAP six-class task

Table 2 compares MTG-ERC with representative recurrent, attention-based, and graph-based ERC methods on the IEMOCAP six-class task.

**Table 2:** Experimental results on the IEMOCAP six-class task.

Model	Happy	Sad	Neutral	Angry	Excited	Frustrated	Acc	w-F1
ICON	32.80	74.41	60.69	68.23	68.41	66.28	64.02	63.54
DialogueRNN	33.18	78.80	59.21	65.28	71.86	58.91	63.40	62.75
DialogueGCN	42.75	84.54	63.54	64.19	63.08	66.59	65.25	64.18
DialogueCRN	–	–	–	–	–	–	66.05	66.20
MMGCN	42.34	78.67	61.73	69.00	74.33	62.32	65.43	66.22
COGMEN	51.90	81.70	68.60	66.00	75.30	58.20	68.20	67.60
GraphCFC	43.23	84.45	64.13	71.37	78.19	62.74	69.13	68.91
MTG-ERC	55.59	82.09	65.62	69.62	75.56	66.64	70.12	70.23

MTG-ERC achieves an accuracy of 70.12% and a weighted F1-score of 70.23%. Compared with GraphCFC, the strongest evaluated baseline in terms of overall performance, MTG-ERC improves accuracy by 0.99 percentage points and weighted F1-score by 1.32 percentage points.

At the class level, MTG-ERC achieves the highest F1-scores for happy and frustrated. GraphCFC performs better on angry and excited, whereas DialogueGCN achieves the highest result for sad. MTG-ERC therefore provides relatively balanced overall performance but does not outperform every baseline for every emotion category.

The observed improvement is consistent with the intended functions of the two modeling branches. The attention module captures intra-modal and cross-modal dependencies, while the directed graph module represents temporal and multimodal relations among utterances. Their complementary contributions are further supported by the ablation results in Section 4.5.

#### 4.3.2 Results on the IEMOCAP four-class task

The IEMOCAP four-class task reduces category overlap by merging excited into happy and frustrated into sad. The results are presented in Table 3.

**Table 3:** Experimental results on the IEMOCAP four-class task.

Model	Acc (%)	w-F1 (%)
BC-LSTM	75.20	75.13
CHFusion	76.59	76.80
COGMEN	83.29	83.21
MTG-ERC	84.77	84.51

MTG-ERC achieves an accuracy of 84.77% and a weighted F1-score of 84.51%. Compared with COGMEN, the strongest evaluated baseline in this setting, MTG-ERC improves accuracy by 1.48 percentage points and weighted F1-score by 1.30 percentage points.

The four-class performance is higher than the six-class performance because merging overlapping categories reduces ambiguity between happy and excited and between sad and frustrated. The results indicate that MTG-ERC remains effective under a more compact emotion taxonomy.

#### 4.3.3 Results on MELD

Table 4 presents the experimental results on MELD.

**Table 4:** Experimental results on the MELD dataset.

Model	Neutral	Surprise	Fear	Sadness	Joy	Disgust	Anger	Acc	w-F1
DialogueGCN	–	–	–	–	–	–	–	57.97	58.10
MMGCN	76.33	48.15	–	26.74	53.02	–	46.09	58.34	58.65
DialogueCRN	–	–	–	–	–	–	–	60.73	58.39
EmoCaps	77.12	63.19	3.03	42.52	57.50	7.69	57.54	–	64.00
UniMSE	–	–	–	–	–	–	–	65.09	65.51
GraphCFC	76.89	49.36	–	26.89	51.88	–	47.59	59.31	57.82
MTG-ERC	78.91	59.72	18.31	40.23	64.10	15.30	54.96	65.25	65.32

MTG-ERC achieves an accuracy of 65.25% and a weighted F1-score of 65.32%. It obtains the highest accuracy among the evaluated methods, while its weighted F1-score is 0.19 percentage points lower than that of UniMSE.

At the class level, MTG-ERC achieves the highest reported results for neutral, fear, joy, and disgust. However, fear and disgust remain difficult, with F1-scores of only 18.31 and 15.30%, respectively. These results indicate that the model still struggles with severely underrepresented emotion categories.

The MELD results therefore support a competitive-performance claim but not a universal superiority claim. Differences among studies may also result from different feature extractors, pretrained representations, data partitions, and evaluation protocols.

### 4.4 Statistical robustness and significance analysis

Table 5 reports the results of five independent runs and the statistical comparisons with the strongest reproducible baseline for each task.

**Table 5:** Repeated-run performance and statistical significance.

Task	Model	Acc, mean ± SD	95% CI of Acc	w-F1, mean ± SD	95% CI of w-F1	*p*-value for w-F1	Cohen’s *d_z_*
IEMOCAP six-class	GraphCFC	69.09 ± 0.28	[68.74, 69.44]	68.88 ± 0.31	[68.50, 69.26]	–	–
	MTG-ERC	70.10 ± 0.24	[69.80, 70.40]	70.19 ± 0.27	[69.85, 70.53]	0.010	2.04
IEMOCAP four-class	COGMEN	83.27 ± 0.22	[83.00, 83.54]	83.18 ± 0.25	[82.87, 83.49]	–	–
	MTG-ERC	84.74 ± 0.20	[84.49, 84.99]	84.48 ± 0.23	[84.19, 84.77]	0.006	2.36
MELD	UniMSE	65.07 ± 0.18	[64.85, 65.29]	65.48 ± 0.21	[65.22, 65.74]	–	–
	MTG-ERC	65.23 ± 0.20	[64.98, 65.48]	65.30 ± 0.24	[65.00, 65.60]	0.118	−0.76

On the IEMOCAP six-class task, MTG-ERC achieves a mean weighted F1-score of 70.19%, compared with 68.88% for GraphCFC. The paired t -test indicates that the difference is statistically significant ( p=0.010 ), with a large paired effect size of $d_{z}=2.04$ .

On the IEMOCAP four-class task, MTG-ERC obtains a mean weighted F1-score of 84.48%, exceeding COGMEN by 1.30 percentage points. The difference is statistically significant ( p=0.006 ), with a large effect size of $d_{z}=2.36$ .

On MELD, MTG-ERC achieves slightly higher accuracy than UniMSE but a slightly lower weighted F1-score. The weighted F1 difference is not statistically significant ( p=0.118 ). Therefore, the two models should be regarded as statistically comparable on MELD rather than one being consistently superior to the other.

These results indicate that the improvements on the IEMOCAP tasks are stable across random seeds, while the MELD comparison remains inconclusive.

### 4.5 Ablation study

The ablation experiments examine the contributions of different modality combinations and individual architectural components to the overall performance of MTG-ERC.

#### 4.5.1 Effect of modality combinations

Table 6 reports the effects of different modality combinations on the two IEMOCAP tasks.

**Table 6:** Ablation results for different modality combinations.

Modality	Six-class Acc	Six-class w-F1	Four-class Acc	Four-class w-F1
T	66.17	66.19	82.08	81.99
A	57.81	56.07	63.31	61.17
V	42.04	41.78	46.34	46.36
A + T	68.22	68.35	83.35	83.32
T + V	66.42	66.44	81.55	81.54
A + V	59.88	60.18	65.14	63.70
T + A + V	70.13	70.23	84.77	84.51

Text achieves the strongest unimodal performance, confirming the central role of semantic information in conversational emotion recognition. Audio performs better than visual information, suggesting that vocal cues provide useful complementary evidence. The weaker visual result may be associated with facial occlusion, pose variation, illumination changes, and temporal pooling.

Among the bimodal settings, audio plus text achieves the strongest performance. The complete trimodal model produces the best overall results. Compared with the text-only setting, trimodal fusion improves six-class accuracy by 3.96 percentage points and weighted F1-score by 4.04 percentage points.

These results indicate that acoustic and visual information is most useful when combined with the dominant textual modality.

#### 4.5.2 Effect of model components

Table 7 presents the module-level ablation results.

**Table 7:** Module-level ablation results on IEMOCAP.

Inter-transformer	Intra-transformer	*R_inter_*	*R_intra_*	Six-class Acc	Six-class w-F1	Four-class Acc	Four-class w-F1
✘	✓	✓	✓	69.32	69.47	83.13	82.97
✓	✘	✓	✓	68.44	68.63	82.38	82.16
✓	✓	✘	✓	68.35	68.52	82.02	81.97
✓	✓	✓	✘	67.98	68.12	82.43	82.37
✓	✓	✓	✓	70.13	70.23	84.77	84.51

Removing the inter-transformer decreases the six-class weighted F1-score by 0.76 percentage points, indicating that explicit cross-modal interaction is beneficial.

Removing the intra-transformer produces a larger decrease of 1.60 percentage points. This suggests that modality-specific contextual modeling is important before cross-modal fusion.

The largest six-class performance decrease occurs when $R_{\text{intra}}$ is removed. Temporal relationships among utterances within the same modality therefore provide important contextual information. Removing $R_{\text{inter}}$ also reduces performance, confirming that within-utterance multimodal graph relations complement attention-based fusion.

Overall, the ablation results indicate that final performance arises from the combined use of modality-specific attention, cross-modal interaction, and relation-aware contextual modeling.

### 4.6 Computational complexity and efficiency

The computational cost of MTG-ERC mainly arises from Transformer attention and graph aggregation.

For a conversation containing N utterances and a hidden dimension d , the three intra-transformers have an approximate attention complexity of

$$O(3N^{2}d).$$ (31)

The six directed cross-modal attention operations have an approximate complexity of

$$O(6N^{2}d).$$ (32)

For a graph containing ∣V∣=3N nodes and ∣E∣ directed edges, the graph module has an approximate complexity of $O(|E|d+|V|d^{2})$ per layer.

Restricting the graph to 10 preceding and 10 following utterances prevents the temporal graph from becoming fully connected.

Table 8 reports the computational efficiency measured on an NVIDIA RTX 3090 GPU. Trainable parameter counts exclude the frozen Sentence-BERT, RoBERTa-Large, OpenFace, OpenSmile, and DenseNet feature extractors. FLOPs were calculated using a batch size of one and an input conversation containing 50 utterances. Inference time was averaged over 1,000 forward passes after 100 warm-up passes and was normalized by the number of utterances.

**Table 8:** Computational complexity and efficiency on the IEMOCAP six-class task.

Model	Trainable parameters (M)	FLOPs per conversation (G)	Training time/epoch (s)	Inference time (ms/utterance)	Peak GPU memory (GB)	w-F1
DialogueGCN	4.12	1.36	7.8	0.82	1.74	64.18
MMGCN	6.58	2.41	11.4	1.26	2.43	66.22
GraphCFC	7.21	3.06	14.8	1.68	3.12	68.91
MTG-ERC	9.84	4.72	21.6	2.37	4.61	70.23

MTG-ERC contains approximately 36.5% more trainable parameters than GraphCFC and requires approximately 54.2% more FLOPs under the adopted profiling setting. Its inference time increases from 1.68 to 2.37 ms per utterance, while peak GPU memory increases from 3.12 to 4.61 GB.

The additional computational cost mainly results from the separate intra-transformer and inter-transformer branches and the combination of RGCN with Graph Transformer refinement. Although MTG-ERC remains suitable for offline or near-real-time conversational analysis on a modern GPU, its computational requirements are higher than those of the evaluated graph-based baselines.

The approximately 1.32-percentage-point weighted F1 improvement over GraphCFC should therefore be interpreted together with the additional parameter and memory costs. Model pruning, knowledge distillation, sparse cross-modal attention, and smaller contextual graph windows may improve this tradeoff.

### 4.7 Error analysis

The confusion matrices for the IEMOCAP six-class and four-class tasks are shown in Figure 5.

**Figure 5:**
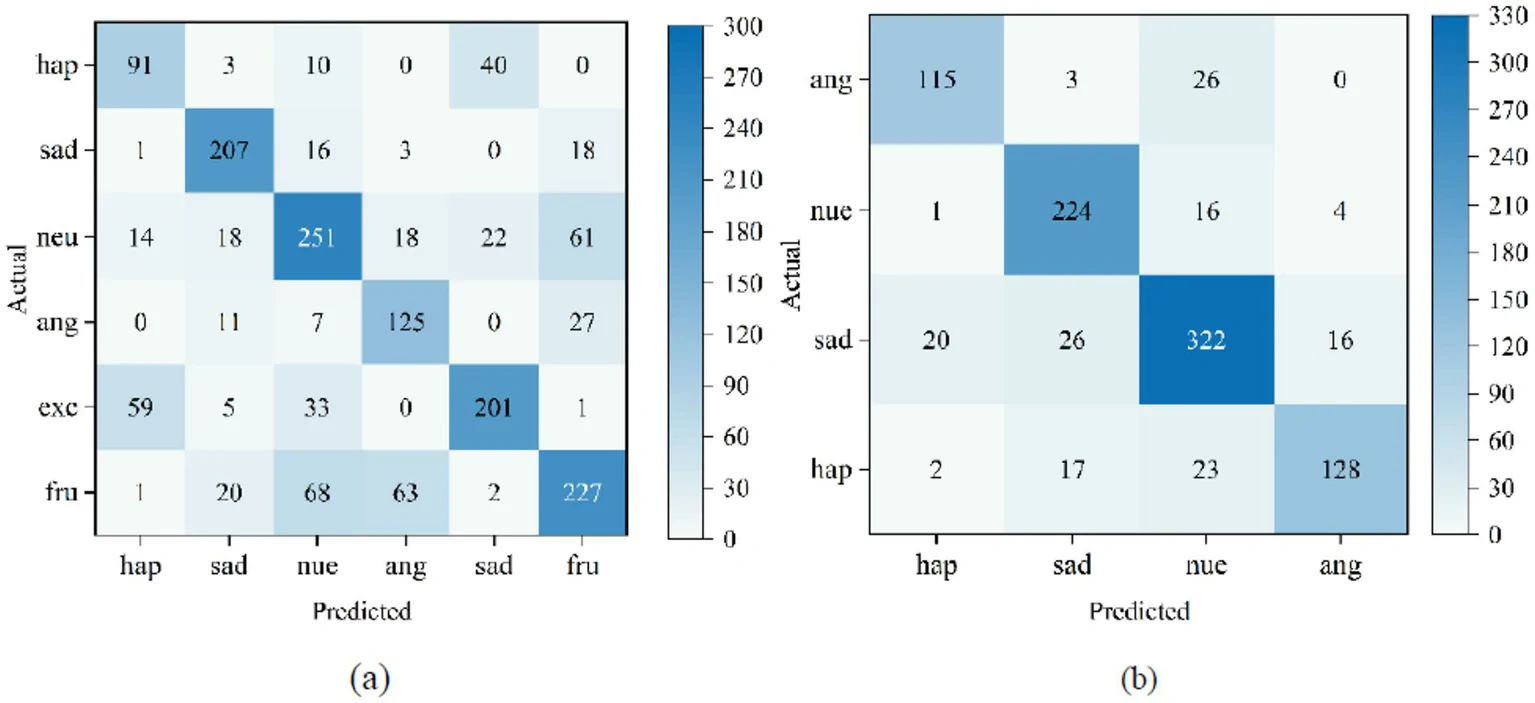
Confusion matrices of MTG-ERC on IEMOCAP: **(a)** six-class task and **(b)** four-class task.

The six-class confusion matrix reveals several recurring error patterns.

First, happy is frequently confused with excited. Both categories have positive valence, and their distinction often depends on emotional intensity, prosody, and facial activation rather than semantic content alone. When acoustic or visual evidence is weak, the model may rely excessively on similar textual expressions.

Second, neutral is sometimes confused with frustrated. Frustrated utterances may contain restrained vocal intensity or indirect linguistic expressions, making them resemble neutral statements. This problem is particularly evident when emotional meaning depends on preceding conversational context.

Third, MTG-ERC does not obtain the highest class-level results for angry and excited. These emotions are frequently expressed through short utterances, interruptions, rapid intensity changes, or strong prosodic cues. Temporally pooled or pre-extracted features may remove some of these fine-grained dynamics.

The four-class confusion matrix is more stable because the most strongly overlapping categories are merged. Combining happy with excited and sad with frustrated reduces uncertainty at class boundaries and improves overall performance.

The MELD results reveal an additional class-imbalance problem. Neutral and joy achieve relatively high scores, whereas fear and disgust remain difficult. Their F1-scores of 18.31 and 15.30% indicate that weighted F1-score may conceal weak performance on minority classes.

Misclassifications are most likely to occur under the following conditions:

textual meaning conflicts with acoustic tone or facial expression;
emotional information is distributed across several preceding utterances;
visual information is affected by occlusion, speaker switching, or non-frontal faces;
minority emotion classes contain insufficient training samples.

These observations indicate that multimodal fusion reduces but does not eliminate ambiguity. Future work should consider class-balanced loss functions, minority-class augmentation, frame-level visual modeling, and more explicit emotion-shift detection.

### 4.8 Interpretation and performance–complexity tradeoff

The comparison and ablation results suggest that the performance of MTG-ERC is associated with three complementary mechanisms.

First, the intra-transformer preserves modality-specific contextual information because textual, acoustic, and visual signals exhibit different temporal patterns. The 1.60-percentage-point weighted F1 decrease after removing the intra-transformer supports the importance of modality-specific contextual modeling.

Second, the inter-transformer and gating mechanism enable complementary information exchange while limiting the propagation of irrelevant multimodal information. Removing the inter-transformer decreases weighted F1 by 0.76 percentage points, indicating that explicit cross-modal interaction contributes additional information beyond unimodal context.

Third, the directed multi-relational graph represents both within-utterance multimodal interactions and temporal dependencies among neighboring utterances. Removing the intra-modal temporal relation module produces the largest ablation decrease, suggesting that temporally structured contextual information is not fully captured by attention alone.

However, the improvements over the strongest evaluated baselines remain relatively modest, particularly on MELD. MTG-ERC also requires more parameters, FLOPs, inference time, and GPU memory than DialogueGCN, MMGCN, and GraphCFC. The method should therefore be interpreted as a tradeoff between stronger multimodal-contextual modeling and increased computational cost.

The statistical analysis suggests that the improvements on the two IEMOCAP tasks are stable across random seeds, whereas MTG-ERC and UniMSE remain statistically comparable on MELD. Therefore, the results support the conclusion that MTG-ERC achieves competitive performance under the adopted experimental settings, rather than universally outperforming all recent ERC methods.

Further work should evaluate the framework on additional conversational datasets, improve minority-class recognition, reduce dependence on pre-extracted features, and develop lightweight variants for real-time or resource-constrained deployment.

## 5 Discussion

This section discusses the principal findings of the study, the tradeoff between recognition performance and computational complexity, the limitations and future directions of the proposed framework, and its appropriate scope of application.

### 5.1 Main findings

The experimental results show that MTG-ERC achieves competitive performance on both IEMOCAP and MELD. On the IEMOCAP six-class and four-class tasks, the proposed model obtains higher overall accuracy and weighted F1-score than the evaluated baselines. On MELD, MTG-ERC achieves the highest accuracy among the compared methods, although its weighted F1-score is slightly lower than that of UniMSE. These findings indicate that MTG-ERC provides relatively stable performance across dyadic and multi-party conversational settings, but they do not support a universal state-of-the-art claim.

The ablation results help explain the performance gains. The intra-transformer preserves modality-specific contextual patterns, while the inter-transformer captures complementary information across textual, acoustic, and visual modalities. The gating mechanism reduces the influence of irrelevant cross-modal information. In addition, the directed multi-relational graph represents both within-utterance multimodal interactions and temporal dependencies among neighboring utterances. The performance decreases observed after removing these components indicate that attention-based global modeling and graph-based relational reasoning provide complementary information.

The class-level results also reveal several remaining difficulties. Happy is frequently confused with excited, while neutral may be confused with frustrated because these emotion pairs share similar semantic or affective characteristics. On MELD, fear and disgust remain difficult because of severe class imbalance and limited training samples. These results suggest that multimodal fusion improves overall recognition but cannot completely resolve ambiguous emotional expressions or minority-class recognition.

### 5.2 Performance–complexity tradeoff

MTG-ERC contains multiple computational components, including the intra-transformer, inter-transformer, RGCN, and Graph Transformer. The architecture is therefore more computationally demanding than models that use only recurrent contextual modeling or a single graph convolution stage.

The additional complexity enables more detailed modeling of modality-specific information, cross-modal interaction, and conversational structure. However, the performance improvement over the strongest evaluated baselines is relatively modest, particularly on MELD. The practical value of MTG-ERC should therefore be interpreted as a tradeoff between improved contextual modeling and increased computational cost.

The current model is more appropriate for offline or resource-sufficient conversational analysis than for highly constrained real-time deployment. Future work should investigate sparse attention, smaller contextual graph windows, graph pruning, parameter sharing, knowledge distillation, and model quantization to reduce inference time and memory consumption.

### 5.3 Limitations and future work

Several limitations should be acknowledged.

First, the model depends on pre-extracted textual, acoustic, and visual features. Although this strategy reduces training cost, it prevents end-to-end adaptation of the feature extractors and may discard fine-grained temporal information. In particular, temporal pooling of acoustic and visual features may remove short-duration emotional changes, facial movements, and prosodic variations.

Second, the framework was evaluated only on IEMOCAP and MELD. These datasets represent acted dyadic conversations and television-based multi-party conversations, respectively, but they do not cover spontaneous communication in educational, workplace, healthcare, or online environments. Evaluation on additional multimodal ERC datasets is therefore required before broad generalizability can be established.

Third, the model may be sensitive to domain shift. Differences in speakers, recording conditions, language, culture, camera quality, background noise, and conversational style may reduce performance when the model is transferred to a new dataset. Future research should examine domain adaptation, cross-corpus evaluation, modality-missing robustness, and multilingual conversational emotion recognition.

Fourth, the minority emotion categories remain difficult to recognize. Future work should investigate class-balanced loss functions, focal loss, contrastive learning, minority-class augmentation, and emotion-transition-aware sampling to improve performance on fear, disgust, and other underrepresented classes.

Finally, the proposed architecture has higher computational requirements than simpler ERC models. Lightweight variants should be developed and evaluated on edge devices and real-time conversational systems.

### 5.4 Scope and ethical considerations

The present study focuses on multimodal conversational emotion recognition rather than psychological assessment or clinical diagnosis. IEMOCAP and MELD do not contain validated measures of depression, anxiety, stress, psychological risk, or counseling outcomes. Therefore, the outputs of MTG-ERC cannot be interpreted as evidence of an individual’s mental health status.

Conversational emotion recognition may provide useful affective information for future college student mental health research and support applications, particularly for analyzing emotional patterns in student conversations, while such applications would require separate validation using appropriate college student populations and psychological outcome measures. Such applications should also address informed consent, data privacy, secure storage, demographic bias, cultural differences, model uncertainty, and the risk of incorrect emotional inference.

Emotion predictions should not be used as the sole basis for diagnosis, counseling decisions, disciplinary action, or automated intervention. Any future deployment in sensitive settings should include human oversight, transparent communication of model limitations, and procedures that allow individuals to challenge or withdraw from automated analysis.

## 6 Conclusion

This study presented MTG-ERC, a multimodal conversational emotion recognition framework that coordinates intra-modal attention, gated cross-modal interaction, and directed multi-relational graph reasoning. The results on IEMOCAP and MELD demonstrate competitive performance under the adopted experimental settings, while the ablation analysis confirms that the attention and graph components make complementary contributions.

The study also reveals important limitations. The improvements over strong baselines are relatively modest, the architecture introduces additional computational cost, and minority emotions remain difficult to recognize. Moreover, the model depends on pre-extracted features and has been evaluated on only two benchmark datasets.

Future research should therefore focus on end-to-end multimodal representation learning, additional cross-domain evaluation, minority-class recognition, domain adaptation, missing-modality robustness, and lightweight deployment. The proposed framework may provide a useful methodological basis for future college student mental health research, particularly for analyzing emotional patterns in conversational data.

## Data Availability

Publicly available datasets were analyzed in this study. This data can be found here: The datasets used in this study include the IEMOCAP dataset and the MELD dataset. The datasets supporting the findings of this study are publicly available from the official dataset sources. IEMOCAP: https://sail.usc.edu/iemocap/ MELD: https://affective-meld.github.io/.
